# Challenges in proteogenomics: a comparison of analysis methods with the case study of the DREAM proteogenomics sub-challenge

**DOI:** 10.1186/s12859-019-3253-z

**Published:** 2019-12-20

**Authors:** Tara Eicher, Andrew Patt, Esko Kautto, Raghu Machiraju, Ewy Mathé, Yan Zhang

**Affiliations:** 1 Department of Computer Science and Engineering, The Ohio State University, Columbus, OH 43210 USA; 2 Department of Biomedical Informatics, College of Medicine, The Ohio State University, Columbus, OH 43210 USA; 3 The Ohio State University Comprehensive Cancer Center (OSUCCC – James), Columbus, OH 43210 USA

**Keywords:** Proteogenomics, mRNA, Random forests, Fuzzy logic, Bayesian networks

## Abstract

**Background:**

Proteomic measurements, which closely reflect phenotypes, provide insights into gene expression regulations and mechanisms underlying altered phenotypes. Further, integration of data on proteome and transcriptome levels can validate gene signatures associated with a phenotype. However, proteomic data is not as abundant as genomic data, and it is thus beneficial to use genomic features to predict protein abundances when matching proteomic samples or measurements within samples are lacking.

**Results:**

We evaluate and compare four data-driven models for prediction of proteomic data from mRNA measured in breast and ovarian cancers using the 2017 DREAM Proteogenomics Challenge data. Our results show that Bayesian network, random forests, LASSO, and fuzzy logic approaches can predict protein abundance levels with median ground truth-predicted correlation values between 0.2 and 0.5. However, the most accurately predicted proteins differ considerably between approaches.

**Conclusions:**

In addition to benchmarking aforementioned machine learning approaches for predicting protein levels from transcript levels, we discuss challenges and potential solutions in state-of-the-art proteogenomic analyses.

## Background

### Proteogenomics and its challenges

Proteogenomics is a field that utilizes genomic and transcriptomic data in conjunction with proteomic data to draw correlations between genes and proteins. While High-Throughput Sequencing (HTS) technologies have commoditized the generation of genomic and transcriptomic data, proteomics still lags behind in both scope and cost due to technological limitations. Assays such as Reverse Phase Protein Arrays (RPPA) [1] have made it possible to quantitate larger numbers of proteins at a time, but they rely on antibody specificity and currently do not exist on a whole-proteome level. Mass spectrometry (MS)-based technologies have also gained prevalence in proteomic research, although still facing limitations in repeatability of identification and consistency of quantification [2]. As protein expression levels are often of interest for biological and biomedical researchers, there is significant interest in developing approaches that would allow a broader spectrum of proteins to be easily and effectively quantified for both diagnostic and research purposes. Therefore, there is an incentive to build models that use mRNA gene expression from HTS assays to predict protein expression levels [3]. Ideally, such approaches would be able to take in data from targeted HTS panels, whole exome or whole genome sequencing, or whole-transcriptome mRNA sequencing, and use the expression levels of one or more genes to predict the corresponding protein expression levels accurately. These models would provide insights into how different levels of biological signals correlate with each other and what dynamic ranges these signals fall into [4].

A limitation to the use of proteogenomics for protein level prediction is the complexity of the human proteome. A review by Kendrick et al. found that there is often limited correlation between mRNA transcript and protein expression levels [5]. Many factors likely contribute to the low correlation, including cell-specific expression patterns, post-translational modifications, and the complex microenvironments of cells, in which many mRNA-mRNA, mRNA-protein, and protein-protein interactions regularly occur. However, the fact that weak correlations do exist between associated transcripts and proteins opens the possibility of purely data-driven prediction of protein levels from transcript levels, which we explore in this paper.

### Case study: NCI-CPTAC DREAM proteomics challenge

The NCI-CPTAC DREAM challenge was organized to collaboratively develop robust methodologies to use the biological relationships between genes and proteins to address challenges in the field of proteogenomic data analysis. For this case study, genomic, transcriptomic, proteomic, and phosphoproteomic data were provided in tumor and adjacent normal tissue pairs of breast and ovarian cancer patients to promote the development of new strategies for proteogenomic data analysis. The DREAM Challenge consisted of 3 related machine-learning proteogenomic sub-challenges, of which we focused on the second: using transcriptomic and DNA copy number information to predict missing protein values in the same sample. The data used in our analysis was given as part of the challenge and is available with a Synapse account.

The contestants of this sub-challenge applied a variety of approaches, but documentation for each consists primarily of method descriptions on the challenge’s wiki page, found at https://www.synapse.org/#!Synapse:syn11522015/wiki/496744. The winning approaches included biologically-inspired ensemble methods (Li, H., unpublished), LASSO with relevant genes selected from existing networks and databases (Kim, S. et al., unpublished), random forests with input filtered by KEGG and PPI pathway association (Yu, H. et al., unpublished), and random forests on codon count, GC content, and folding energy with pathway analysis and patient clustering (Park, S. et al., unpublished). Specifically, the top team included transcript levels and excluded CNV as features. They combined single gene - single protein predictors with random forest models built on all transcripts. Other contestants also applied other methods, such as additional random forest approaches (Chen, J. et al., unpublished), spline regression (Narsapuram, V. et al., unpublished), network models (Li, Y. et al., unpublished), genetic models (Belmadani, M., unpublished), linear regression (Fóthi, Á. et al., unpublished), elastic net (Afyounian, E. et al., unpublished) (Lau, E. and Lam,M., unpublished), and neural networks (Kambara, Y. and Okuda,S., unpublished). We note that some methods included additional data not provided by the organizers of the challenge: protein-protein interaction networks, biological pathways, codon count, GC content, and folding energy. The top team (Li, H., unpublished) attempted to filter transcripts using GO terms, but they found that it decreased performance.

### Choice of models

Rather than making use of the additional biological knowledge as described above (PPI networks, shared pathways, etc.), our work focuses on the comparison of methods for data-driven analysis alone. Understanding the utility of each method can assist researchers in choosing an appropriate method for prediction on their data set, especially with limited data. In addition, the relationship between transcript and protein abundance remains largely uncharacterized, and the performance of each model can lead to a fuller understanding of the underlying biological process driving the abundance of each protein.

We critically analyzed and compared the performance of purely data-driven methods in a unified, comparable setting (e.g., same training/test sets, same performance measurement). We compared the following methods for addressing this sub-challenge: Bayesian Networks (BN), random forests (RF), LASSO, and fuzzy logic predictors. These methods were chosen because they represent different classes of models, all of which are used in applied machine learning and make different assumptions about the relationship between features and outcome (in our case, transcript and protein abundance). Differences between these methods are summarized in Table 1. LASSO assumes that protein abundance is a sparse linear function of transcript abundance [6], which may or may not be true. The fuzzy logic model does not assume linearity but assumes that protein abundance is determined by fuzzy set operations, in our case, the intersection of possible abundances from each transcript. While we are not aware of any other uses of fuzzy logic in this exact manner, fuzzy logic has been used in other bioinformatic applications. Barbosa et al. use a similar fuzzy intersection technique to infer distribution of amphibian species [7]. In addition, fuzzy sets are used to model gene product similarity between genes and secondary protein structure in literature [8].

**Table 1:** Contrasting Features of the Methods

Model	Nonlinear interactions	Computationally efficient	Probabilistic model
LASSO		x	
Random Forests	x	x	
Fuzzy Logic	x		x
Bayesian Networks	x		x

Contrasting features of the different algorithms used to predict protein levels from transcripts. “Nonlinear interactions” indicates that the algorithm does not assume that protein levels are a linear function of transcript levels. “Computationally efficient” means that predictions were able to be made in less than 12 h on the Ohio Supercomputer Center (OSC) cluster using all transcripts in the data as input. “Probabilistic model” means that the predictions are given as probabilities, representing uncertainty in the data

RF models make neither of the assumptions of the aforementioned models and can be used to model dependency chains and correlations between variables [9], but interpreting feature importance using RFs is not straightforward [10] and RFs can be prone to overfitting [11]. BNs are designed to capture conditional relationships, but can be heavily dependent on the choice of prior distribution and may fail to resolve conflicting relationships in the data [12]. We benchmarked these approaches in a unified framework.

### Characteristics of the data set

The data used for this study consists of two data sets, one for breast cancer (BRCA) and one for ovarian cancer (OVA). These data were derived from TCGA and CPTAC consortia. Each data set is comprised of DNA-based copy number data, transcript and protein expression data. Microarray data is included for OVA, but we do not use it in our analyses. This is because we wished to focus on a pan-cancer approach and to ensure consistency between BRCA and OVA data, and microarray data was only available for the OVA data set. Transcript data were generated with RNA-seq, and have been median-aggregated on a per-sample level before RSEM Z-score transformation. This results in normalized expression values that have a mean of 0 and a standard deviation of 1 per sample. Despite the sample-level normalization, the gene-level expression was also relatively normalized, with the majority of expression centered around a value of 0 and with a relatively subdued standard deviation (Additional file 1: Figure S1). Protein expression data was generated using mass spectrometry-based iTRAQ (isobaric tag for relative and absolute quantitation) [13], which uses reporter molecules to return a ratio of abundance of protein in paired samples. We used the *scale* function in R to perform normalization both for protein abundance and transcripts across the BRCA and OVA, and combined data sets.

The OVA protein samples underwent quantification at two different institutes (JHU and PNNL), resulting in two data sets. We examined the correlation between protein abundance levels in OVA from the JHU and PNNL data sets to determine whether the two data sets could be integrated in a straightforward manner in our analyses. These plots (Additional file 1: Figure S2) illustrate that the data distributions are correlated but not identical. We combined the data from both institutes by retaining only proteins measured in the OVA datasets of both institutes. Thus, our final OVA data set contained the intersection of the OVA from both JHU and PNNL.

**Fig. 1:**
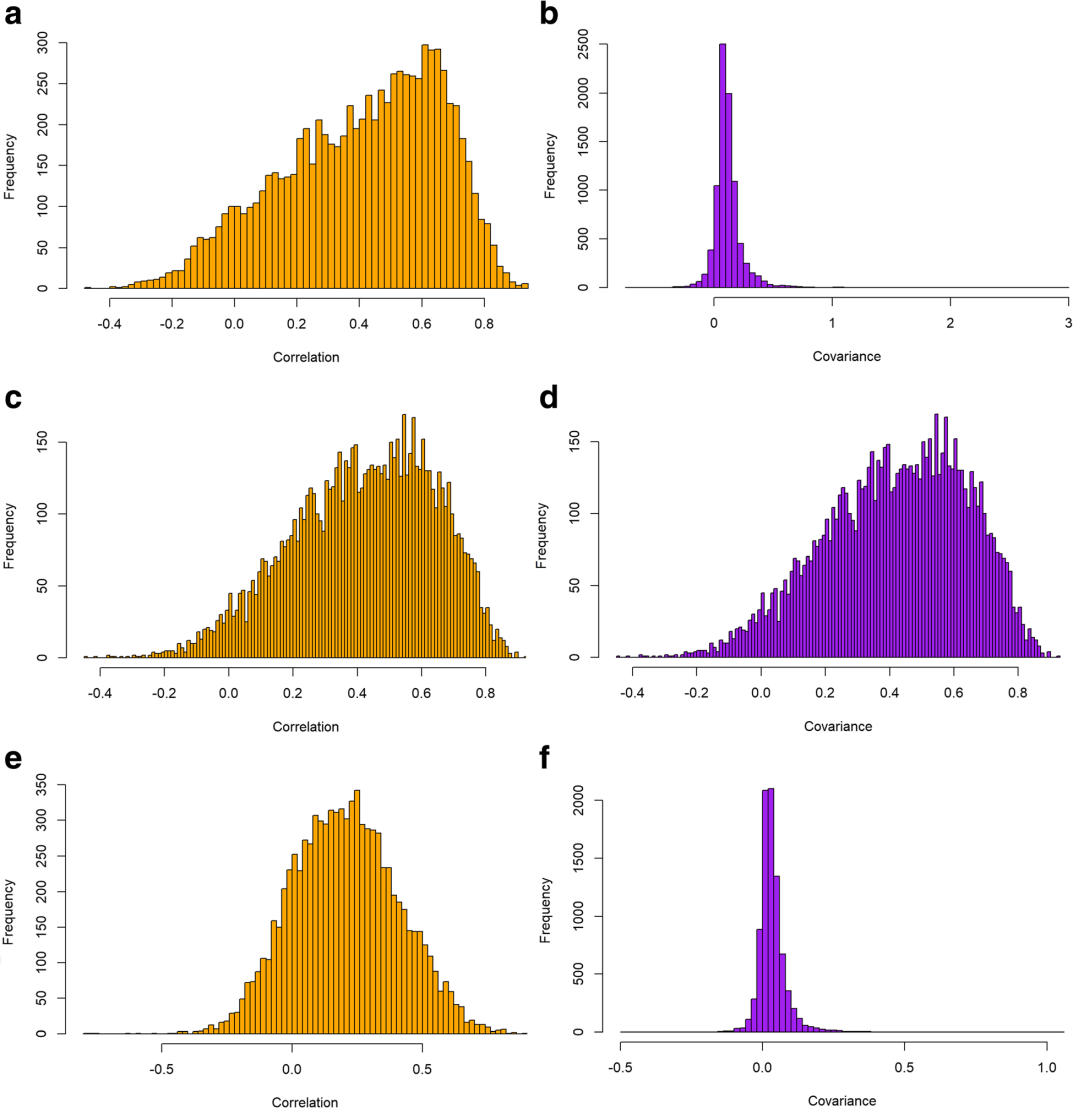
Protein, CNV, and mRNA Covariances. Histograms of (**a**) correlations between BRCA CNV and mRNA (**b**) covariances between BRCA CNV and mRNA (**c**) correlations between BRCA mRNA and proteins (**d**) covariances between BRCA mRNA and proteins (**e**) correlations between BRCA CNV and proteins (**f**) covariances between BRCA CNV and proteins

We also examined the distribution of correlations between CNV, transcripts, and proteome measurements to assess the extent of global correlations between each data type (Fig. 1). These distributions reflect findings from other previous studies, which have suggested that gene-protein correlation (Spearman’s correlation coefficient) tends to hover around 0.47, on average [14, 15]. An analysis of the covariances was even more stark, with only mRNA-protein showing any notable covariances. This lack of relationship between transcripts and copy numbers presents a potential challenge when using CNV or transcript abundance to predict protein abundance. It is notable that, while both CNV and transcript abundance exhibit correlation to protein abundance, transcript exhibits higher correlation on average. Given our observations and the fact that transcriptomic levels have been shown to associate more closely with protein levels than DNA copy number in previous studies [16–18], we focused on the use of transcript levels to predict protein levels. We therefore only utilized the transcript data to benchmark machine learning approaches to predicting protein abundances. This is consistent with the approach used by the DREAM challenge winning team (Li, H., personal communication). Of the data sets available, the BRCA MS/MS iTRAQ proteomic data, BRCA RNA-seq data, OVA JHU LC-MS/MS iTRAQ proteomic data, and OVA transcripts were selected. Only proteomic/transcriptomic data taken from the same samples were considered for the study.

## Results

The goal of our study was to explore the feasibility of using a purely data-driven approach to predict protein abundance using mRNA levels and to compare data-driven approaches used therein. All of our approaches were tested on the same data sets using the same benchmarking setup for the purpose of direct comparison.

### Bayesian networks

**Fig. 2:**
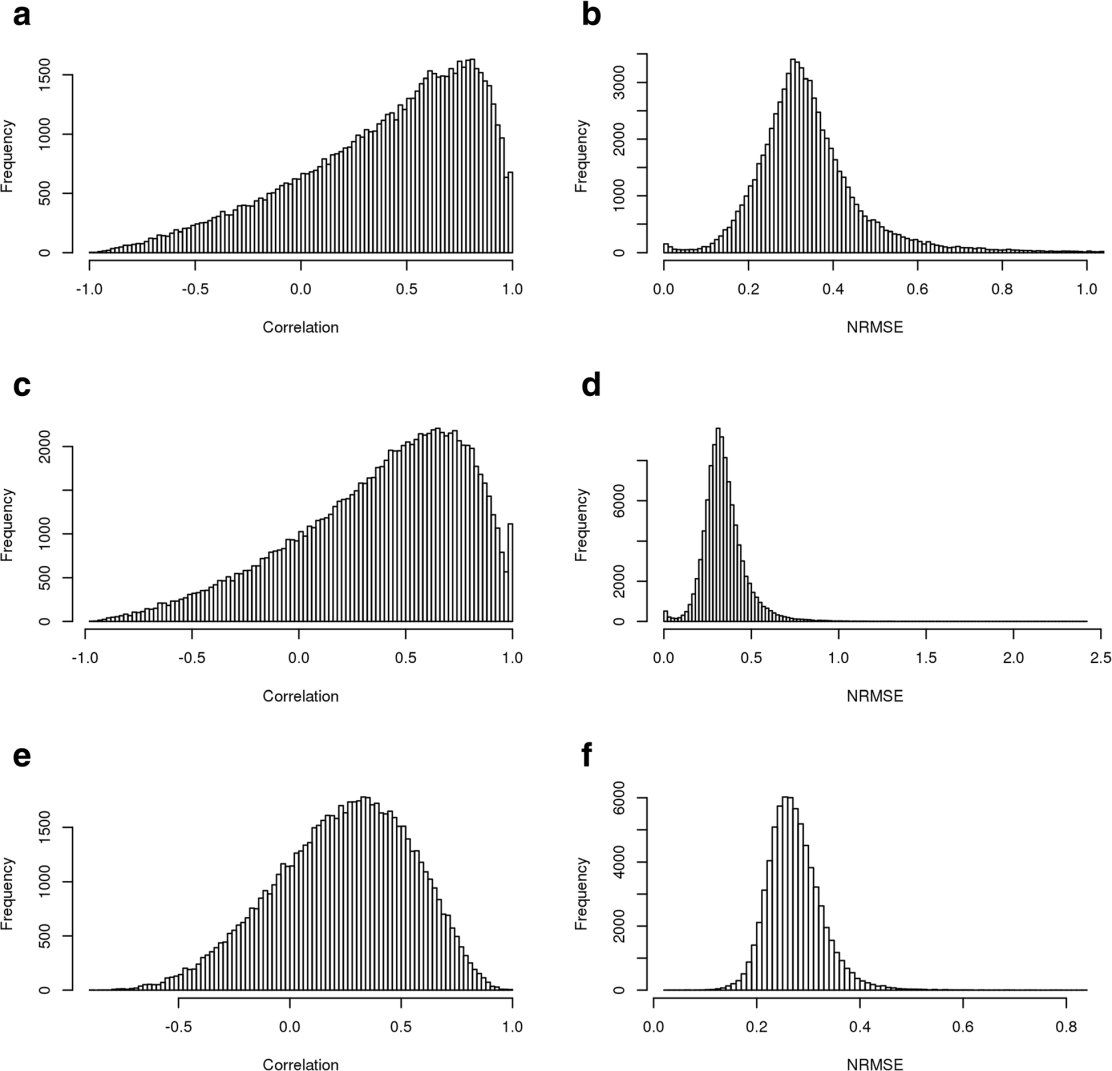
Results of Bayesian Network Prediction. Histograms of (**a**) correlations between ground truth and predictions for combined data (**b**) NRMSE (Normalized Root Mean Squared Error) of predictions for combined data (**c**) correlations between ground truth and predictions for BRCA (**d**) NRMSE of predictions for BRCA (**e**) correlations between ground truth and predictions for OVA (**f**) NRMSE of predictions for OVA

The results of the BN method are displayed in Fig. 2. We tested 9 different algorithms included in the *bnlearn* package in R, and found that ARACNE provided the fewest missing predictions with a comparable prediction accuracy to other BN inference algorithms. On the combined BRCA and OVA data, we obtained a median correlation of 0.237 across all ten cross-validations between predictions and ground truth and an NRMSE of 0.274, with no failed predictions. On BRCA data only, we obtained a median correlation of 0.376 and an NRMSE of 0.344, with no failed predictions. On OVA data only, we obtained a median correlation of 0.397 and an NRMSE of 0.361, with 1 failed prediction. Results are summarized in Table 2. Table 3 lists the 10 proteins found in the highest number of top 100 lists, along with the number of cross-validations for which these proteins reached the top 100 list. This allows us to evaluate the consistency of the predictions across models built using each cross-validation; having the same proteins represented in the top 100 list across multiple cross-validations indicates that the method is generating consistent models for these proteins across cross-validations.

**Table 2:** Bayesian Network Correlation and NRMSE

Dataset	Median Correlation	Median NRMSE
Combined	0.237	0.274
Breast	0.376	0.344
Ovarian	0.397	0.361

The NRMSE and correlation results for the Bayesian network model using the optimal ARACNE structural inference algorithm

**Table 3:** Best-Predicted Proteins using Bayesian Networks

Combined		BRCA		OVA	
Protein Name	Count	Protein Name	Count	Protein Name	Count
CMBL	9	BCAN	9	CBX2	9
WFDC2	8	MAEL	9	GFOD2	9
SERPINB3	8	MAGEC1	9	GP1BA	9
NUCB2	8	TESC	9	SERPINB3	8
MAGEA9	8	WIPF3	9	SLC35B1	8
FDXR	8	CD1A	8	CRADD	7
SFX2	7	HLA-DQB2	8	CYP11A1	7
RABEP1	7	AKR1B15	7	MINDY2	7
MYH14	7	CST4	7	MMP10	7
ALCAM	7	CYP4F22	7	CALB1	6

The list of proteins whose predictions are most frequently found to have one of the top 100 correlation values to ground truth across 10 cross-validations. These predictions are generated using the Bayesian network model

### Fuzzy logic prediction

**Fig. 3:**
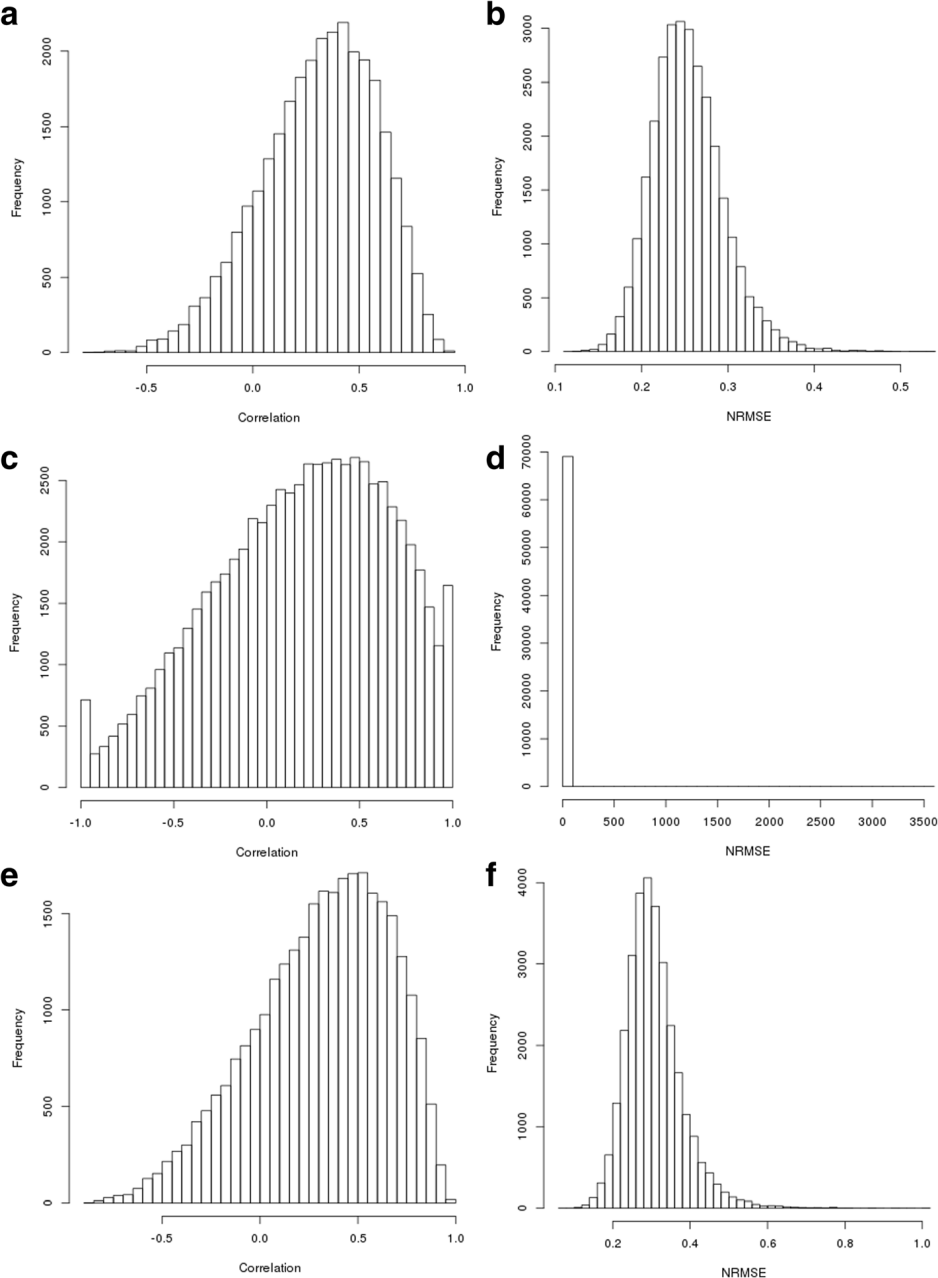
Results of Fuzzy Logic Prediction. Histograms of (**a**) correlations between ground truth and predictions for combined data (**b**) NRMSE of predictions for combined data (**c**) correlations between ground truth and predictions for BRCA (**d**) NRMSE of predictions for BRCA (**e**) correlations between ground truth and predictions for OVA (**f**) NRMSE of predictions for OVA

We predicted the abundance of each protein using fuzzy logic predictors as described in the Methods section. We found that the optimal tuning parameters differed between the combined, BRCA, and OVA models. The optimal parameters were **τ** = 1 and **α** = 0.1 for the combined model, **τ** = 1 and **α** = 0.3 for the BRCA model, and **τ** = 0.5 and **α** = 0.1 for the OVA model as shown in Table 4. On the combined data, we obtained a median correlation of 0.338 across all ten cross-validations between predictions and ground truth and an NRMSE of 0.295. On BRCA data only, we obtained a median correlation of 0.228 and an NRMSE of 0.367. On OVA data only, we obtained a median correlation of 0.355 and an NRMSE of 0.297. When we examine the distribution of correlations for each of these experiments as shown in Fig. 3, we observe similar patterns to those seen in the RF and BN models. The BRCA data has heavier tails than the OVA and combined plots, and the error is slightly lower in the combined plots data. Robustly predicted proteins are summarized in Table 5.

**Table 4:** Fuzzy Logic Correlation and NRMSE

**τ**	**α**	NRMSE (All)	NRMSE (OVA)	NRMSE (BRCA)	Corr (All)	Corr (OVA)	Corr (BRCA)
**0.5**	**0.1**	0.2508628	0.2974011	0.3637616	0.3314102	0.3547708	0.1980717
**0.5**	**0.3**	0.2619791	0.3173626	0.4037628	0.3172434	0.3508759	0.2163652
**1**	**0.1**	0.2503973	0.295076	0.3494831	0.3376378	0.3528844	0.2132373
**1**	**0.3**	0.25587	0.305844	0.3667606	0.3261083	0.3386826	0.2281974
**2**	**0.1**	0.2576439	0.3026524	0.3432097	0.2640732	0.2909315	0.1978028
**2**	**0.3**	0.2602301	0.3078683	0.3484859	0.2540885	0.2700112	0.1973525

The NRMSE and correlation results for the fuzzy logic model with respect to tuning parameters τ and α

**Table 5:** Best-Predicted Proteins using Fuzzy Logic

Combined		BRCA		OVA	
Protein Name	Count	Protein Name	Count	Protein Name	Count
ARHGDIB	6	DLG5	6	CRKL	5
DTX3L	5	PHYHD1	6	FMNL1	5
LCP1	5	AR	5	ISG15	5
PARP14	5	EMC3	5	MX1	5
ACTR3	4	S100A14	5	NNMT	5
CARM1	4	SSH3	5	CNN3	4
COTL1	4	ADIRF	4	EIF4G1	4
DDX60	4	AGR3	4	IFIT1	4
DOCK2	4	CAPN2	4	MARCKS	4
IFIT5	4	CMC2	4	NSFL1C	4

The list of proteins whose predictions are most frequently found to have one of the top 100 correlation values to ground truth across 10 cross-validations. These predictions are generated using the fuzzy logic model

### Random forest regression

We found that the optimal tuning parameter for mtry was 5, as shown in Table 6. On the combined BRCA and OVA set, we obtained a median correlation of 0.489 across all ten cross-validations between predictions and ground truth and an NRMSE of 0.233. On BRCA data only, we obtained a median correlation of 0.357 and an NRMSE of 0.337. On OVA data only, we obtained a median correlation of 0.5561061 and an NRMSE of 0.266.

**Table 6:** Random Forest Correlation and NRMSE

mtry	NRMSE (All)	NRMSE (OVA)	NRMSE (BRCA)	Corr (All)	Corr (OVA)	Corr (BRCA)
5	0.2329111	0.2663645	0.3374966	0.4887368	0.5561061	0.3569502
10	0.2345528	0.2677073	0.3404584	0.4856333	0.5547034	0.3505388
25	0.2344793	0.2684557	0.3398591	0.4849816	0.5539119	0.3539051

**Fig. 4:**
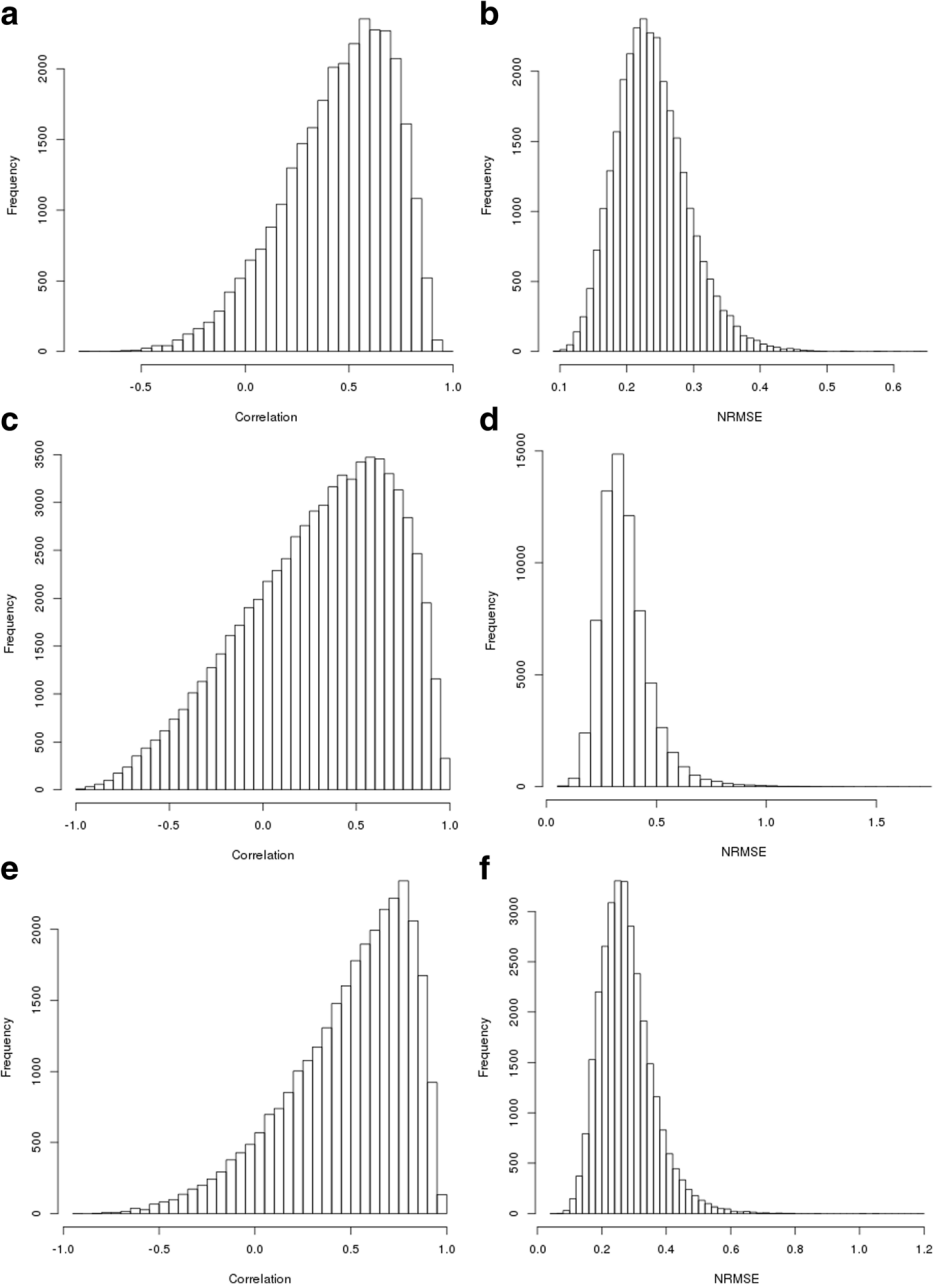
Results of Random Forest Analysis. Histograms of (**a**) correlations between ground truth and predictions for combined data (**b**) NRMSE of predictions for combined data (**c**) correlations between ground truth and predictions for BRCA (**d**) NRMSE of predictions for BRCA (**e**) correlations between ground truth and predictions for OVA (**f**) NRMSE of predictions for OVA

These correlations are clearly skewed right (Fig. 4), indicating that the method is useful for prediction of many proteins. As seen in our other analyses, the tails are heavier for the BRCA data. NRMSE distribution is similar to that seen in the fuzzy logic models.

To analyze overfitting of the models, we extracted the 100 proteins with the highest correlation values within each cross-validation and computed the overlap. The following table lists the 10 proteins found in the highest number of top 100 lists, along with the number of cross-validations for which these proteins reached the top 100 list. Table 7 lists the 10 proteins found in the highest number of top 100 lists, along with the number of cross-validations for which these proteins reached the top 100 list. This table shows more consistency across cross-validations than the fuzzy logic model, but less than the BN.

**Table 7:** Best-Predicted Proteins using Random Forest

Combined		BRCA		OVA	
Protein Name	Count	Protein Name	Count	Protein Name	Count
MAP 1B	6	ERAP2	6	ASRGL1	6
PSIP1	6	GRB7	6	LAP3	6
ACADSB	5	PACSIN2	5	GMPR	5
ANPEP	5	SSH3	5	ICAM1	5
ASRGL1	5	ERBB2	4	MAP 1B	5
CMBL	5	ESR1	4	MSN	5
DDX58	5	IFIT5	4	VCP	5
FAM129A	5	NCAPH	4	WARS	5
HMGCL	5	PPFIA1	4	XPO5	5
OXCT1	5	PRODH	4	ASS1	4

The list of proteins whose predictions are most frequently found to have one of the top 100 correlation values to ground truth across 10 cross-validations. These predictions are generated using the random forests model

### LASSO regression

**Fig. 5:**
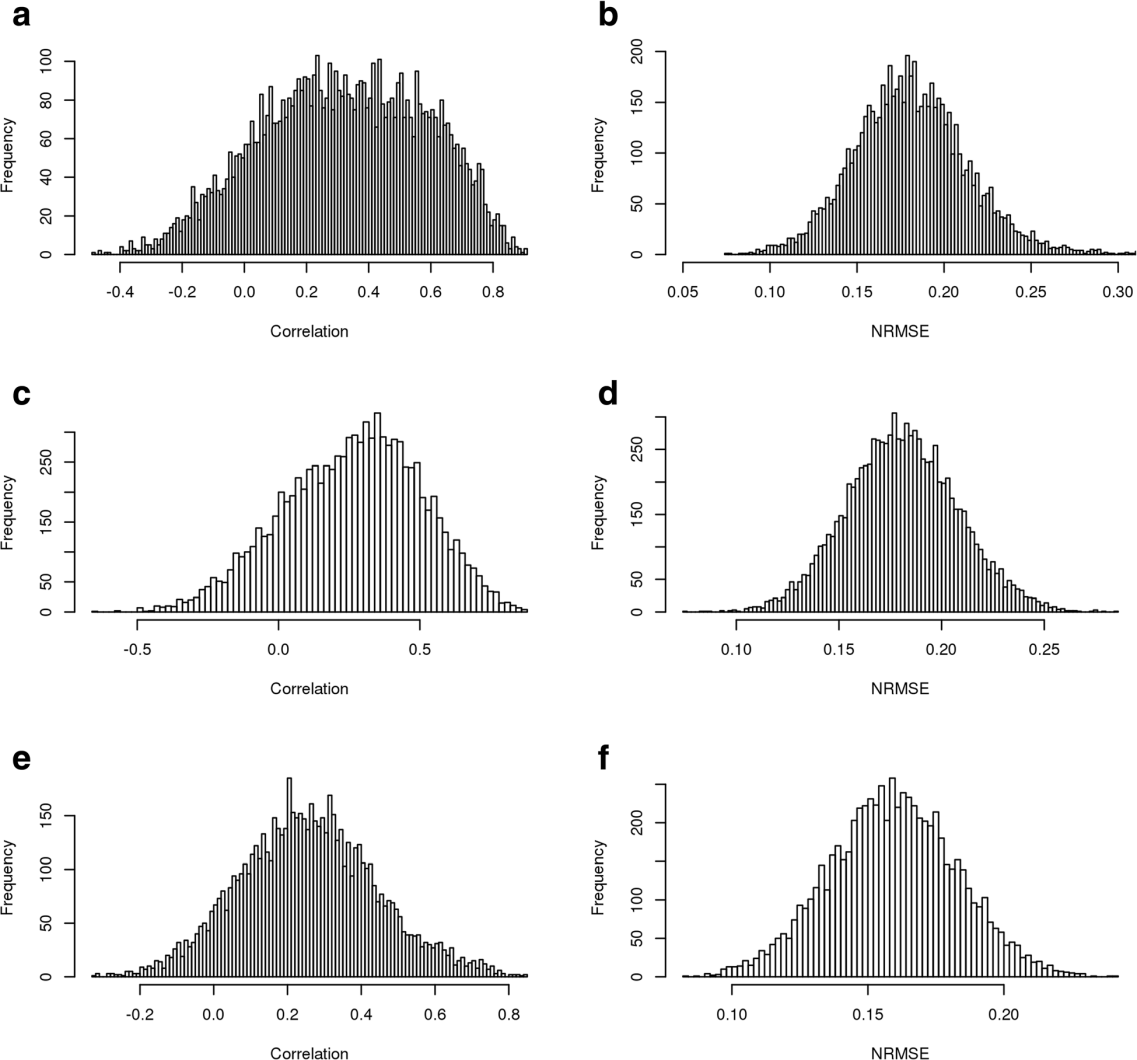
Results of LASSO Analysis. Histograms of (**a**) correlations between ground truth and predictions for combined data (**b**) NRMSE of predictions for combined data (**c**) correlations between ground truth and predictions for BRCA (**d**) NRMSE of predictions for BRCA (**e**) correlations between ground truth and predictions for OVA (**f**) NRMSE of predictions for OVA

The results of the LASSO regression are displayed in Fig. 5. The *caret* package automatically determines the optimal tuning parameters for each LASSO model using a test grid approach. On the combined BRCA and OVA set, we obtained a median correlation of 0.256 across all ten cross-validations between predictions and ground truth and an NRMSE of 0.159, with 4 failed predictions. On BRCA data only, we obtained a median correlation of 0.262 and an NRMSE of 0.182, with 687 missing predictions (6.86% of all proteins). On OVA data only, we obtained a median correlation of 0.317 and an NRMSE of 0.197. These results are summarized in Table 8. The LASSO model performed relatively poorly compared to the other tested methods, perhaps reflective of the complex nonlinear relationships between transcripts and proteins. Table 9 highlights the best-predicted proteins by the LASSO method, which indeed show a high degree of overlap with robustly predicted proteins by other methods, e.g., CMBL (RFs, BNs), WFDC2 (BNs), and ASS1 (RFs). Further, the distribution of LASSO predictions did not exhibit left skewness in their correlation distribution, unlike RF and BN. We therefore found that in these data sets, more sophisticated methods than LASSO were able to achieve significantly better prediction results.

**Table 8:** LASSO Correlation and NRMSE

Dataset	Median Correlation	Median NRMSE
Combined	0.256	0.160
Breast	0.262	0.181
Ovarian	0.317	0.197

The NRMSE and correlation results for the LASSO model using the optimal λ value detected by the caret package

**Table 9:** Best-Predicted Proteins using LASSO

Combined		BRCA		OVA	
Protein Name	PCC	Protein Name	PCC	Protein Name	PCC
CMBL	0.8490182	NUCB2	0.8796467	OXCT1	0.9077667
WFDC2	0.8407794	CRAT	0.8784636	PLAA	0.9068848
ASS1	0.8349155	PREX1	0.8697881	DDX58	0.9059744
INPP4B	0.8283892	HSPA2	0.8610863	C9orf64	0.899744
ALCAM	0.8207547	KANK1	0.858247	ABHD14B	0.8889331
TUBB3	0.8137392	SLC9A3R1	0.8511751	SLC34A2	0.8820199
CRAT	0.8101374	H2AFY2	0.8503076	OAS2	0.8815647
LGALS3	0.8087911	STK39	0.8479941	DHX29	0.8793457
CRABP2	0.8033509	FKBP5	0.8478364	NMNAT1	0.878037
CD109	0.7873721	PHGDH	0.8454966	GBP2	0.8727454

The list of proteins with the highest mean correlation between predicted value and actual value across 10-fold cross-validation. These predictions are generated using the LASSO model. *PCC:* Pearson correlation coefficient

### Ensemble

**Fig. 6:**
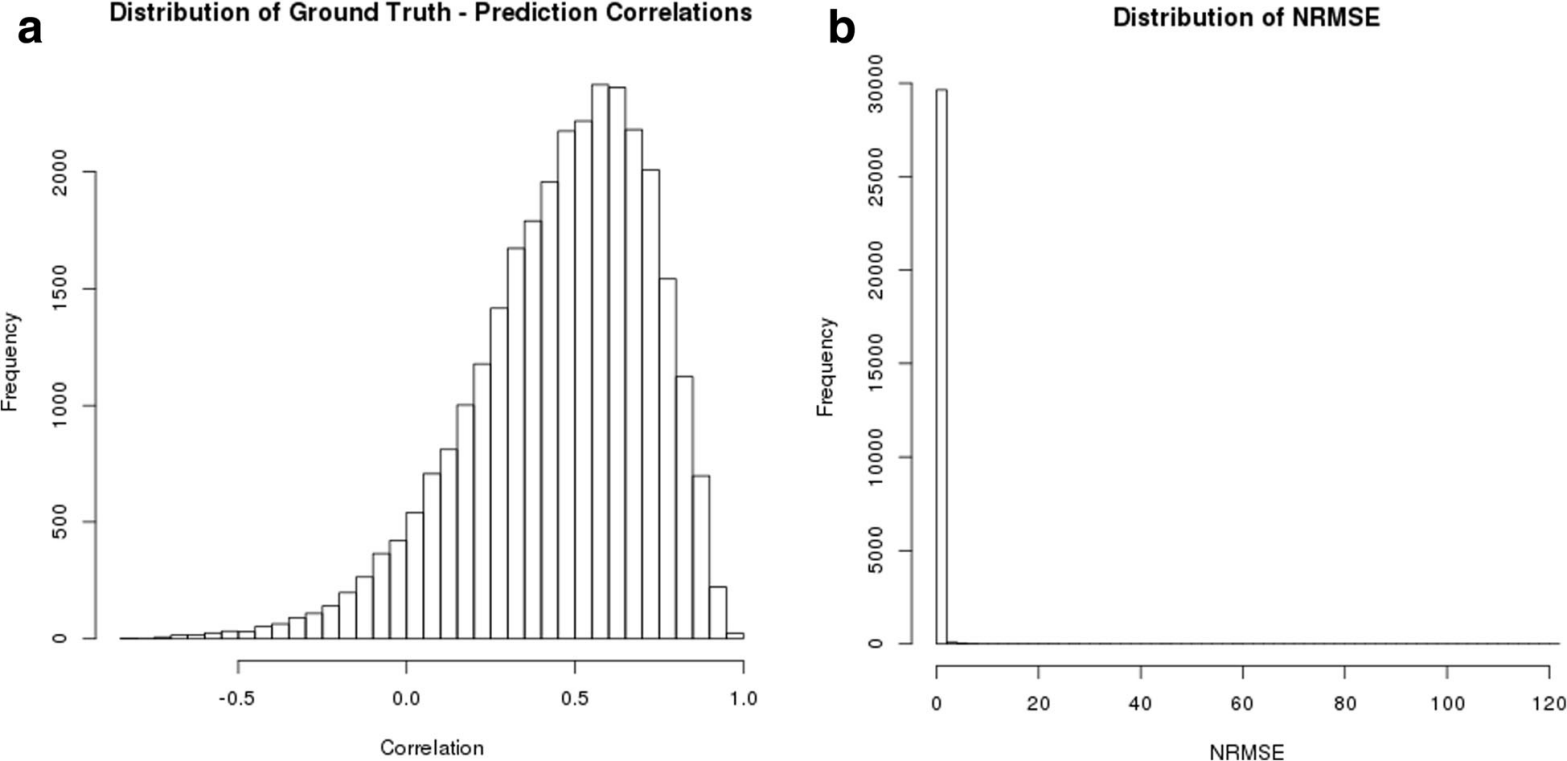
Results of Ensemble Analysis. Histograms of (**a**) correlations between ground truth and predictions for combined data (**b**) NRMSE of predictions for combined data

Our ensemble method combined the predictions of each of the four methods using a weighted sum, where weights for each model and cross-validation were determined by the training accuracies of each of the four methods on that protein and cross-validation. For the ensemble, we evaluated the correlation and NRMSE on the combined BRCA and OVA samples for comparison with our other methods’ results on combined BRCA and OVA samples. We obtained a median correlation value of 0.497 and a median NRMSE value of 0.257. Notably, this correlation value is slightly higher than any of the other models, although RF comes very close in performance. The median NRMSE value is close to that of RF as well. The distribution of correlation values (Fig. 6) is dense near 0.5, with few negative correlation values. From Table 10, the ensemble model appears to be less consistent across multiple cross-validations than the BNs, but slightly more consistent than other models. It is notable that the proteins reported here include proteins reported as consistently well-predicted in other models: NUCB2 and WFDC2 are among the best-predicted proteins by the BN method, CRABP2, LGALS3, and WFDC2 are among the best-predicted by LASSO, and IFIT5 is among the best-predicted by the fuzzy logic method. H2AFY2, while not among the best-predicted list for any of the other models on the combined data, is one of the best-predicted by LASSO when using BRCA data alone.

**Table 10:** Best-Predicted Proteins using the Ensemble

Protein Name	Count
H2AFY2	7
EML2	6
CRABP2	5
LGALS3	5
IFIT5	5
NUCB2	5
BLMH	5
WFDC2	5
CTPS2	4
CAMK2D	4

The list of proteins with the highest mean correlation between predicted value and actual value across 10 cross-validations. These predictions are generated using the ensemble model. *PCC* Pearson correlation coefficient

## Discussion

**Fig. 7:**
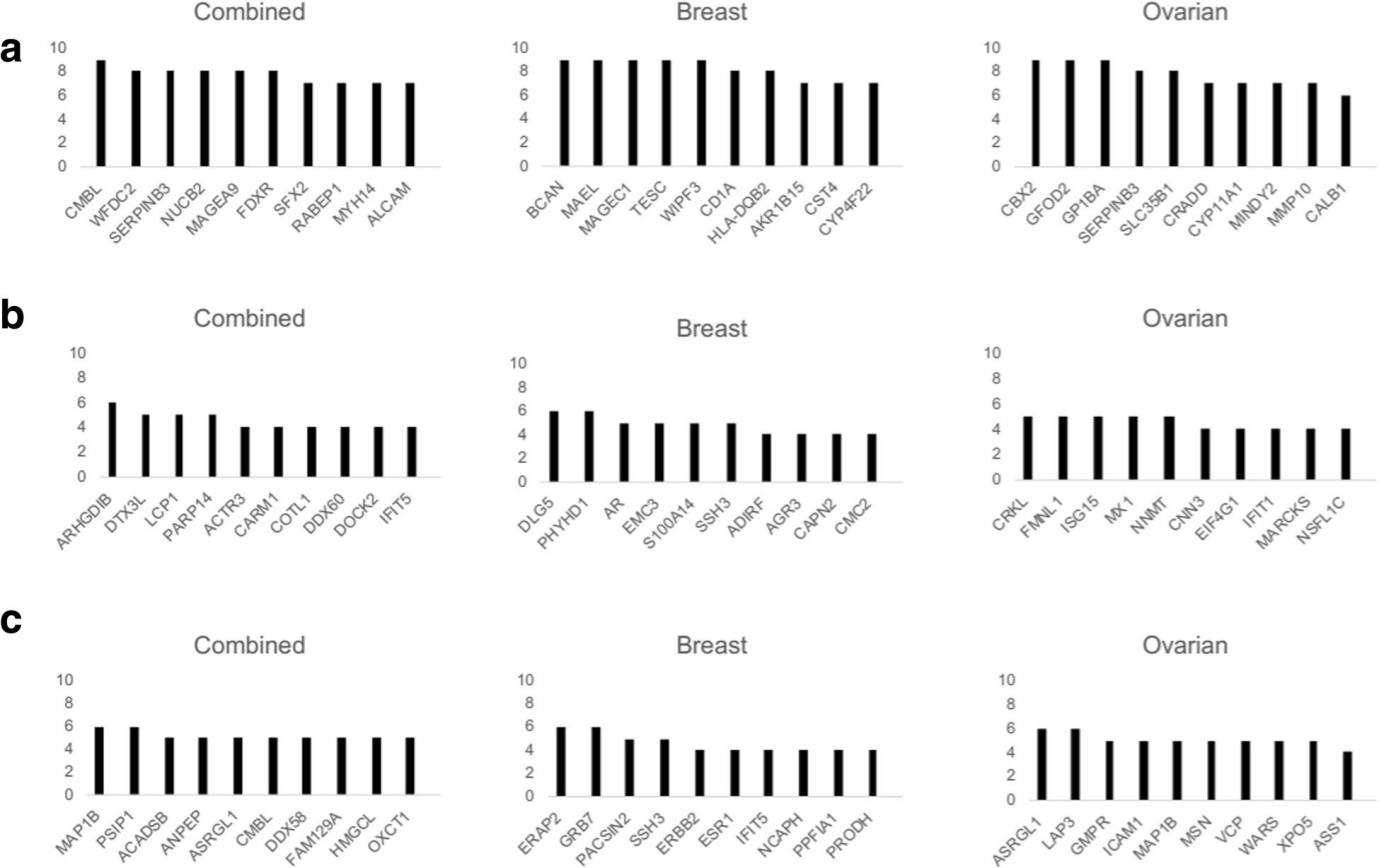
Frequently Well-Predicted Proteins Across Cross-Validations by Dataset and Algorithm. Histograms show the number of times a protein was in the top 100 by correlation coefficient across 10 cross-validations in the (**a**) Bayesian network model, (**b**) fuzzy logic model, and (**c**) random forests model

We applied four different algorithms to predict protein abundance from mRNA in breast (BRCA) and ovarian (OVA) cancer data sets and a combined data set. These methods were chosen to span a variety of different features and drawbacks present in existing methods that are capable of predicting protein levels from transcript levels. Overall, our results indicate that the RF classifier yields the best performance across all proteins. This is primarily evidenced by the median correlation values of 0.49 for BRCA, 0.36 for combined, and 0.55 for OVA; NRMSE was lower in LASSO than in the other three methods. These results can be used to infer possible biological relationships between mRNA and protein abundance. We see that levels of many proteins can be inferred using decision trees built on mRNA values, as long as one can learn the correct decision tree structure. Therefore, we can say that many transcripts of protein abundance are conditionally dependent on the values of other transcripts. BNs also model the phenomenon of conditional dependence and achieve competitive correlation values with RFs. Notably, BNs appear to be the most consistent across cross-validations, at least in terms of the consistency of proteins with the top 100 correlation values (Fig. 7). This offers a potential explanation for the high performance of the network models, as the complex regulation structures between molecules are not likely to be linear. BNs were also notable for their comparable accuracy to other methods while using a truncated list of features as input. The fuzzy logic predictors showed comparable performance, but the proteins for which fuzzy logic performed best were distinct from those for which RFs or BNs performed best. This exemplifies that some proteins have a relationship with their transcripts that is more similar to an AND model than to a conditional dependence model.

Finally, we note that the list of proteins most frequently found to have high correlation across cross-validations differs both between phenotypes and between methods. This observation holds true whether the individual or combined BRCA and OVA datasets were used as input. This interesting finding indicates that, for some proteins, models of conditional dependence are more effective in some phenotypes (i.e., BRCA vs. OVA) than in others. This observation thus implies that a “one model fits all” approach may not be as valuable as combining different types of models. Our ensemble method achieves better results by combining the output from multiple methods, also supporting this conclusion. Further studies to evaluate which specific proteins are modeled best by specific methods are thus warranted.

## Conclusions

We found that data-driven approaches to protein abundance prediction from genes can be effective but also present challenges. (1) One must pay attention to variations in the source of the data (by institute, in this case) and the amount of data available. This is in terms of sample size, missing values, and availability of a genomic data set by patient phenotype (such as microarray data, which was available only for OVA). (2) It is important to choose an appropriate filtering technique due to the dimensionality of the data; prediction using all mRNA would not have been reasonable due to the imbalance between sample count and feature count; for BNs, this approach would have been computationally infeasible as well. While we use correlation for feature selection, other methods for identifying informative transcript/protein relationships could also be applied. (3) One must consider nonlinearity in the choice of a model, and the true model may follow different types of nonlinear functions per protein and phenotype.

## Methods

### Method benchmarking

**Fig. 8:**
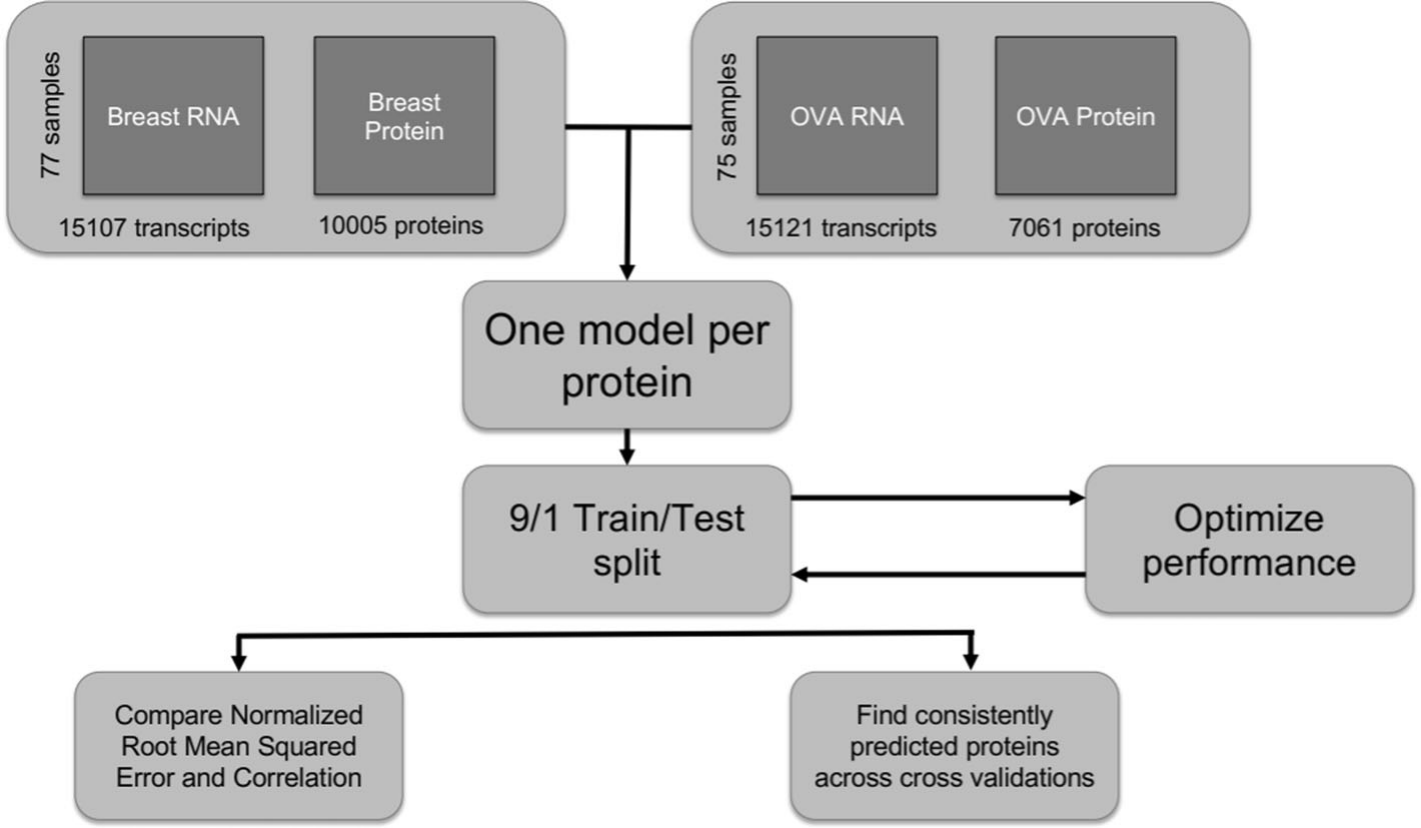
Benchmarking Setup. An overview of our common benchmarking setup for comparing performances of different algorithms in predicting protein levels from transcript levels

An overview of the benchmarking setup used to compare the different methods in the study is provided in Fig. 8. We compared performance within the BRCA and OVA datasets as well as a “combined” dataset which was a concatenation of samples of the two cohorts. For each protein, we built separate models, either using all transcripts for the “computationally efficient” models (LASSO, RF), or a reduced number for the inefficient models (BNs, fuzzy logic). For cross-validation, we randomly split our BRCA, OVA, and combined data sets into 10 partitions. For each cross-validation, we considered 9 of the 10 subsets as training data, and the last subset as validation. As an exhaustive search for all possible configurations is computationally challenging for the fuzzy logic and multivariate BN models, we used correlation to reduce the search space of transcripts to be considered for use in predicting individual protein levels. For each cross-validation, we only considered transcripts that were relatively highly correlated or anti-correlated with the protein of interest in the training data. This heuristic preemptively reduced the number of relationships considered and focused on transcripts that exhibited high correlation with the protein whose levels are being predicted. To this end, global Spearman’s correlations were calculated between each protein and transcript, and those transcripts with the top 8 correlations were chosen for each protein. Preliminary testing showed that for the BN model, reductions in accuracy when removing transcripts outside of the top 8 decreased significantly.

### Fuzzy logic prediction

Fuzzy logic is based on the idea of fuzzy sets [19]. Fuzzy sets are sets in which degrees of membership, rather than absolute membership, exist. Fuzzy logic methods are methods of analysis that make use of fuzzy set operations. While fuzzy logic is often used in classification models, it has also been used in regression by [20, 21]. We use it for the regression task of predicting protein abundance given transcript.

In our approach, the base set of interest is a continuous set of possible predictions of a protein according to a transcript, and the degree of membership of any abundance level in this set is the value of the distribution function of abundance for all samples within a threshold of the transcript value. Once we have obtained our fuzzy sets for each transcript filtered using the preprocessing method described above, we then intersect them. From this, we obtain a final, narrowed degree of membership for each possible abundance level that combines all transcripts. To obtain the final prediction, we select the candidate abundance level (from a continuous distribution) that has the highest membership probability. Intuitively, we choose the abundance value most agreed upon by multiple transcript distributions.

**Fig. 9:**
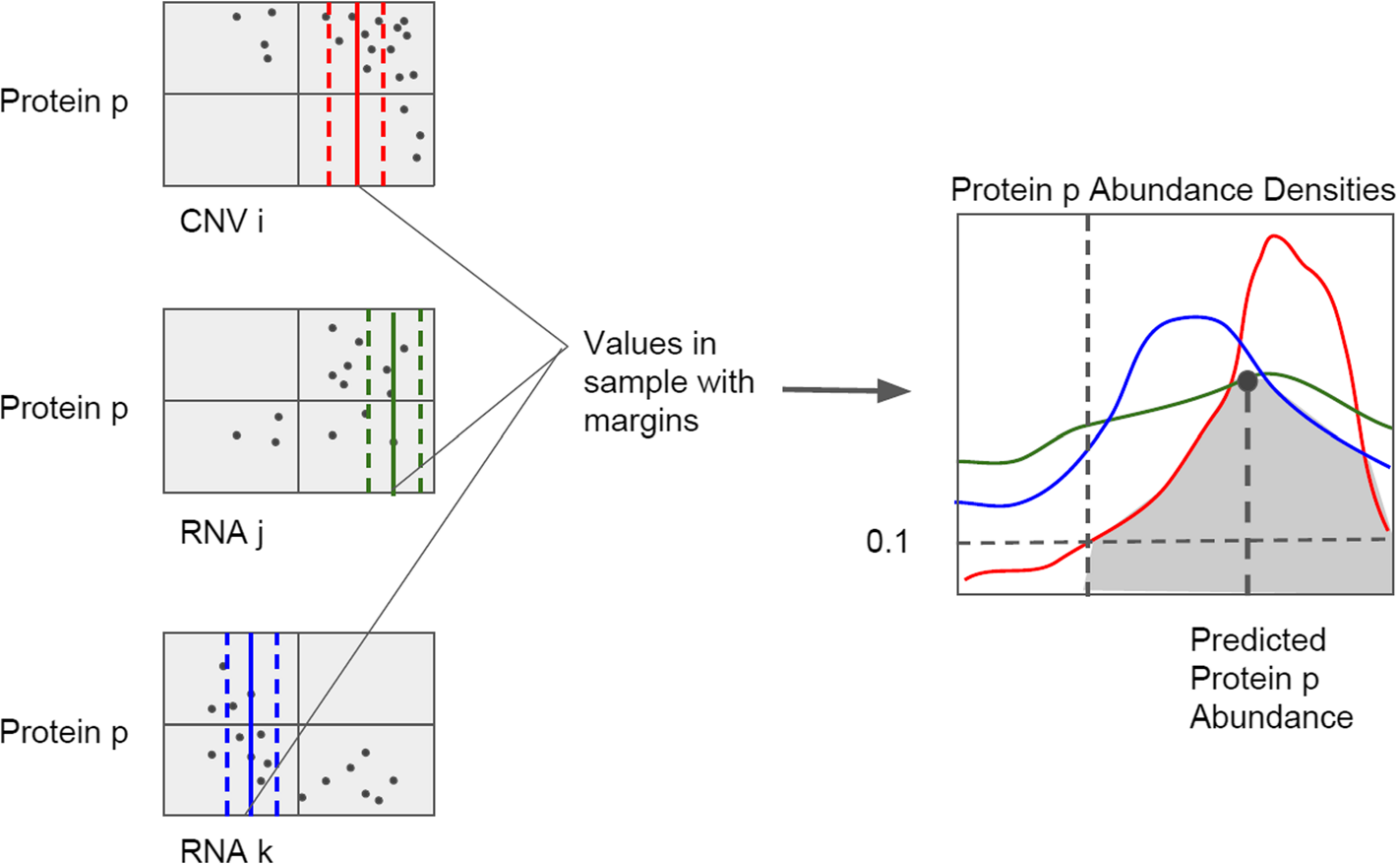
Fuzzy Logic Prediction Procedure. Multiple mRNA loci are selected as influential in the protein abundance by the preprocessing step. For each of these, the distribution of all training samples is stored, and all samples within a margin of +/− 0.1 standard deviations of the value of the sample to predict are used to generate a local distribution. All local distributions are then merged to obtain a final abundance prediction

An overview of this method is provided in Fig. 9. To generate our fuzzy logic models, we first computed a density distribution for each protein with respect to each transcript, using only the values of the transcript within a threshold τ of the current sample. We tested τ = 0.5, 1, and 2 standard deviations. For each transcript, we limited the prediction range to include only those protein levels for which the density was above a threshold of **α**. We tested **α** = 0.1 and 0.3. We also considered a cutoff of 0.5, but many of the density distributions did not contain any values with densities above 0.5, so we do not report this result here. We discarded those transcripts with less than 10 samples above the threshold.

Finally, we combined the density distributions of all transcripts. We considered the prediction range for the protein to be the range shared by all transcripts. Then, we split this range into intervals of 0.1 standard deviations and computed the minimum density among all transcripts at each interval. Our final prediction was the 0.1 standard deviation interval in which this minimum density was maximized. Intuitively, this approach predicts the protein level that is most likely to be found in the training data among all transcripts.

### Multivariate Bayesian networks

BNs are commonly used to elucidate relationships between variables where precise domain knowledge is lacking, with a wide-ranging list of applications in data mining and machine learning [22, 23], psychology [24, 25], and biology [26–28], among others. BNs are advantageous due to their ability to represent conditional dependence between variables in a joint probability distribution, producing a data-driven approximation of relationships between variables. Another advantage is that BNs can perform regression tasks, predicting the most likely value of a random variable of interest (in this case, missing protein abundance) from its parent nodes (transcripts that the protein has detected conditional dependence upon). BNs have been shown to avoid overfitting and work relatively well with low amounts of data compared to other multivariate approaches if an appropriate prior is specified [29, 30], which is advantageous given the small sample sizes of the data sets.

BN approaches require two major steps: a network reconstruction step, in which an algorithm is applied to detect the network structure which maximizes the posterior probability distribution of the data that represent the most likely graphical structure of the underlying joint probability distribution, and a fitting step, in which the joint probability distribution of the network skeleton is calculated, given the conditional dependencies detected.

The *bnlearn* R package was used to construct these BNs, using the ARACNE constraint-based structure learning algorithm. Missing values for transcript or protein levels were imputed by constructing and fitting a network with only complete training samples, and inputting complete observations of parent nodes into the corresponding local probability distribution of the node to be imputed (done using the default “parent” method of the *impute()* function).

### Random forest regression

We created RF models using 100 trees for the BRCA, OVA, and combined data sets respectively, using the *randomForest* package in R. The parameter commonly used to tune RFs is mtry, or the number of variables sampled at each split of a tree in the forest. For our models, we used the *caret* package to tune the parameters.

### LASSO regression

LASSO Regression is a form of linear regression that uses regularization to shrink coefficients that contribute little to the fit of the model, resulting in a more sparse, generalizable model. As LASSO calculations are relatively fast, we were able to include all transcripts without missing values that had nonzero variance as input to the models. We used the *train()* function with the ‘lasso’ method from the *caret* package, with the default search space of λ from 0.1 to 0.9. Each model took every mRNA transcript with no missing values as features. Similar to other methods, a 10-fold cross-validation with a 90/10 training split was used to test model accuracy.

### Ensemble

In addition to evaluating the methods described above on an individual basis, we also combined these models to obtain an ensemble model and used this model to predict the combined BRCA and OVA data. In the ensemble, the LASSO, RF, BN, and fuzzy logic predictions for each protein were combined using a weighted sum of their training accuracies for that protein. The purpose for including the ensemble was to examine the benefit of choosing an optimal model class for each protein, and it is motivated by the expectation that not all proteins will be best modeled by a single model class.

## Supplementary information

**Additional file 1.** Supplementary Figures

## Data Availability

The datasets supporting the conclusions of this article are available in the NCI-CPTAC DREAM Proteogenomics Challenge Synapse repository, https://www.synapse.org/#!Synapse:syn8228304/files/. Code is available on request from the authors.

## Abbreviations

BRCA: DREAM Proteomics Challenge breast cancer data set
CNV: Copy number variation
HTS: High-throughput sequencing
IDH: Isocitrate dehydrogenase
iTRAQ: Isobaric tag for relative and absolute quantitation
JHU: Johns Hopkins University
KEGG: Kyoto Encyclopedia of Genes and Genomes
LASSO: Least absolute shrinkage and selection operator
LC-MS/MS: Liquid chromatography tandem mass spectrometry
mRNA: Messenger RNA
MS/MS: Tandem mass spectrometry
NCI-CPTAC: National Cancer Institute’s Clinical Proteomic Tumor Analysis Consortium
NRMSE: Normalized root mean squared error
OVA: DREAM Proteomics Challenge ovarian cancer data set
PNNL: Pacific Northwest National Laboratory
PPI: Protein-protein interaction network
RPPA: Reverse phase protein arrays

## References

[1] Boellner S, Becker K-F (2015). Reverse phase protein arrays-quantitative assessment of multiple biomarkers in biopsies for clinical use. Microarrays (Basel, Switzerland).

[2] Schubert OT (2017). Quantitative proteomics: challenges and opportunities in basic and applied research. Nat Protoc.

[3] Nesvizhskii AI (2014). Proteogenomics: concepts, applications and computational strategies. Nat Methods.

[4] Mehdi AM (2014). Predicting the dynamics of protein abundance. Mol Cell Proteomics.

[5] Kendrick, N. A gene’s mRNA level does not usually predict its protein level. Available from: https://kendricklabs.com/wp-content/uploads/2016/08/WP1_mRNAvsProtein_KendrickLabs.pdf

[6] Schneider A (2010). Linear regression analysis: part 14 of a series on evaluation of scientific publications. Dtsch Arztebl Int.

[7] Barbosa AM, Real R (2012). Applying fuzzy logic to comparative distribution Modelling: a case study with two sympatric amphibians. Sci World J.

[8] Xu D, Bondugula R, Popescu M, Keller J. Bioinformatics and fuzzy logic. In: 2006 IEEE international conference on fuzzy systems: IEEE; 2006. p. 817–24. https://ieeexplore.ieee.org/document/1681805/authors#authors.

[9] Chen X, Ishwaran H (2012). Random forests for genomic data analysis. Genomics.

[10] Louppe G (2014). Understanding random forests: from theory to practice.

[11] Tang C (2018). When do random forests fail? 32nd Conf. Advances in Neural Information Processing Systems.

[12] Wang P (2004). The limitation of Bayesianism. Artif Intell.

[13] Ross PL (2004). Multiplexed protein quantitation in Saccharomyces cerevisiae using amine-reactive isobaric tagging reagents. Mol Cell Proteomics.

[14] Zhang H (2016). Integrated Proteogenomic characterization of human high-grade serous ovarian Cancer. Cell.

[15] Greenbaum D (2003). Comparing protein abundance and mRNA expression levels on a genomic scale. Genome Biol.

[16] Smolke CD, Keasling JD (2002). Effect of copy number and mRNA processing and stabilization on transcript and protein levels from an engineered dual-gene operon. Biotechnol Bioeng.

[17] Myhre S (2013). Influence of DNA copy number and mRNA levels on the expression of breast cancer related proteins. Mol Oncol.

[18] Liu Y (2016). Leading edge review on the dependency of cellular protein levels on mRNA abundance.

[19] Zadeh LA (1965). Fuzzy Sets. Inf Control.

[20] Yager RR (1982). Fuzzy prediction based on regression models. Inf Sci (Ny).

[21] Real R (2006). Obtaining environmental Favourability functions from logistic regression. Environ Ecol Stat.

[22] Rohekar RY (2018). Constructing deep neural networks by Bayesian network structure learning.

[23] Cheng J, Ell Greiner R (1999). Comparing Bayesian network classifiers. Proceedings of the Fifteenth conference on Uncertainty in artificial intelligence.

[24] Litvinenko A (2017). Application of Bayesian networks for estimation of individual psychological characteristics.

[25] Jacobs RA, Kruschke JK (2011). Bayesian learning theory applied to human cognition. Wiley Interdiscip Rev Cogn Sci.

[26] Needham CJ (2007). A primer on learning in Bayesian networks for computational biology. PLoS Comput Biol.

[27] Isci S (2014). Bayesian network prior: network analysis of biological data using external knowledge. Bioinformatics.

[28] Dong C, Yue H (2016). Identification of functional connections in biological neural networks using dynamic Bayesian networks. IFAC-PapersOnLine.

[29] van de Schoot R (2015). Analyzing small data sets using Bayesian estimation: the case of posttraumatic stress symptoms following mechanical ventilation in burn survivors. Eur J Psychotraumatol.

[30] McNeish D (2016). On using Bayesian methods to address small sample problems. Struct Equ Model A Multidiscip J.

