# Supplementary material for: Challenges in proteogenomics: a comparison of analysis methods with the case study of the DREAM proteogenomics sub-challenge
Source: BMC Bioinformatics. 2019 Dec 20;20(Suppl 24):669. doi: 10.1186/s12859-019-3253-z (PMC6923881; doi:10.1186/s12859-019-3253-z)
Supplement: Supplementary file 1 — Additional file 1. Supplementary Figures [file 12859_2019_3253_MOESM1_ESM.zip › ProteogenomicsReviewSupplementary.pdf]

Supplementary Figures

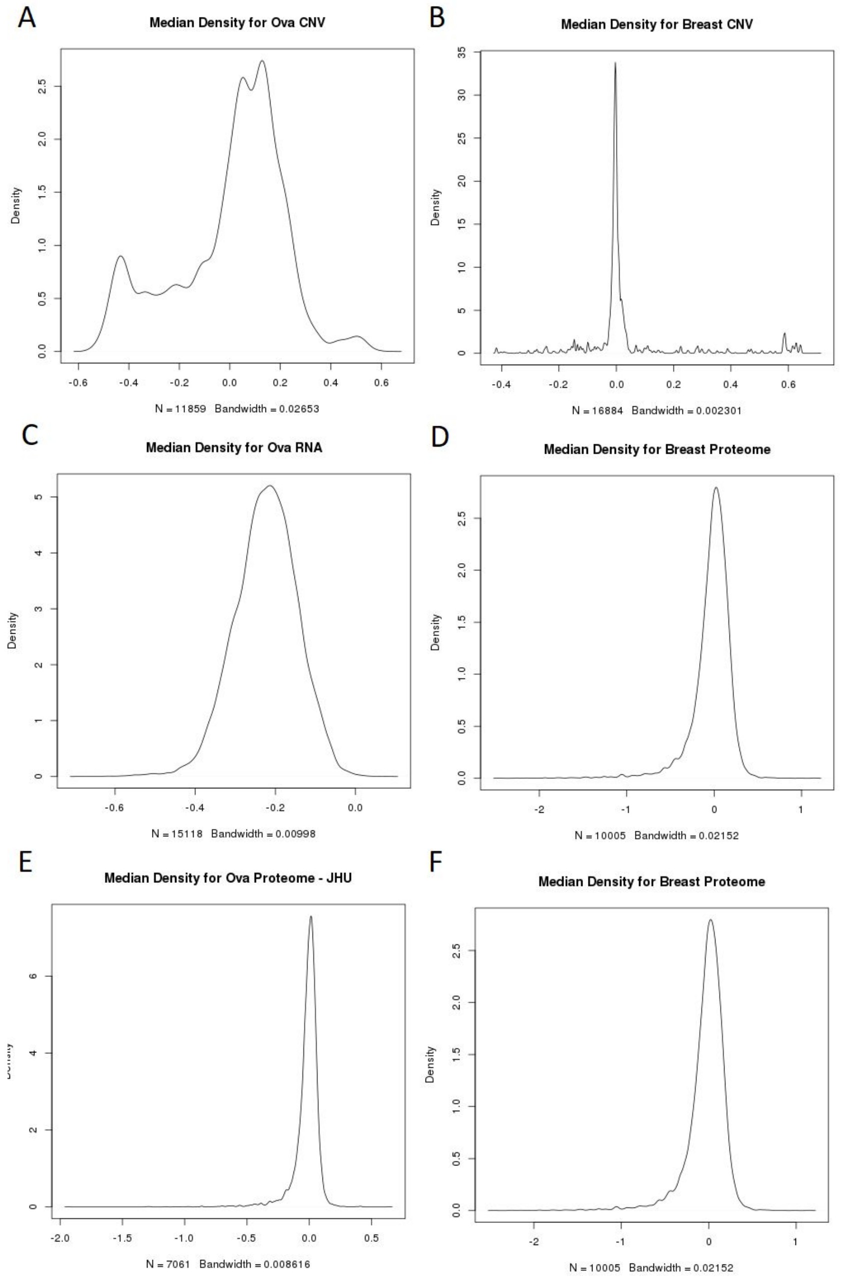

Supplementary Figure 1. (A) Copy number variation distribution for all genes in the ovarian data set (B) Copy number variation distribution for all genes in the breast data set (C) mRNA distribution for all genes in the ovarian data set (D) mRNA distribution for all genes in the breast data set (E) protein abundance distribution for all genes in the ovarian data set (JHU) (F) protein abundance distribution for all genes in the breast data set

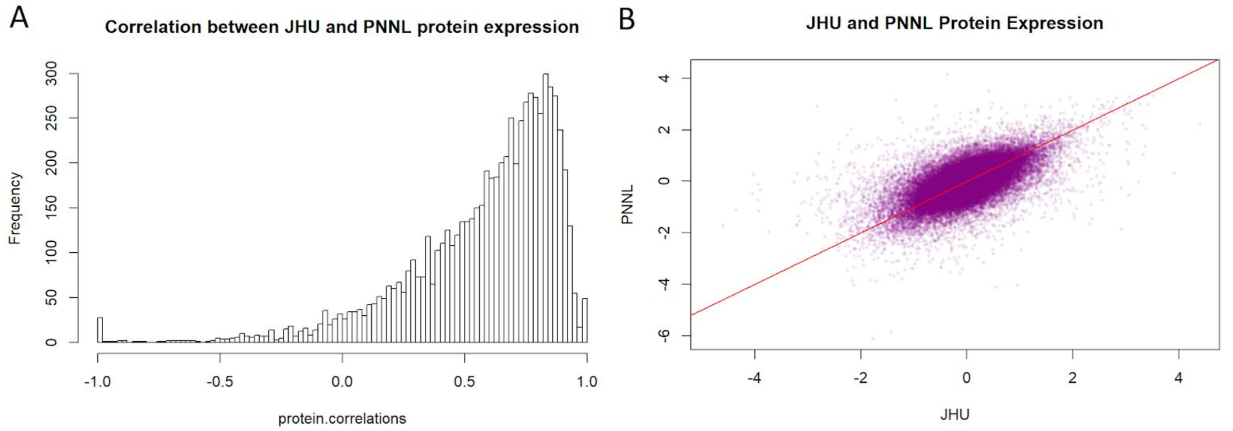

Supplementary Figure 2. (A) The correlation between protein expression measured at JHU and PNNL (B) All protein expression levels measured at JHU and PNNL
