# Supplementary figures and images for: Challenges in proteogenomics: a comparison of analysis methods with the case study of the DREAM proteogenomics sub-challenge
Source: BMC Bioinformatics. 2019 Dec 20;20(Suppl 24):669. doi: 10.1186/s12859-019-3253-z (PMC6923881; doi:10.1186/s12859-019-3253-z)

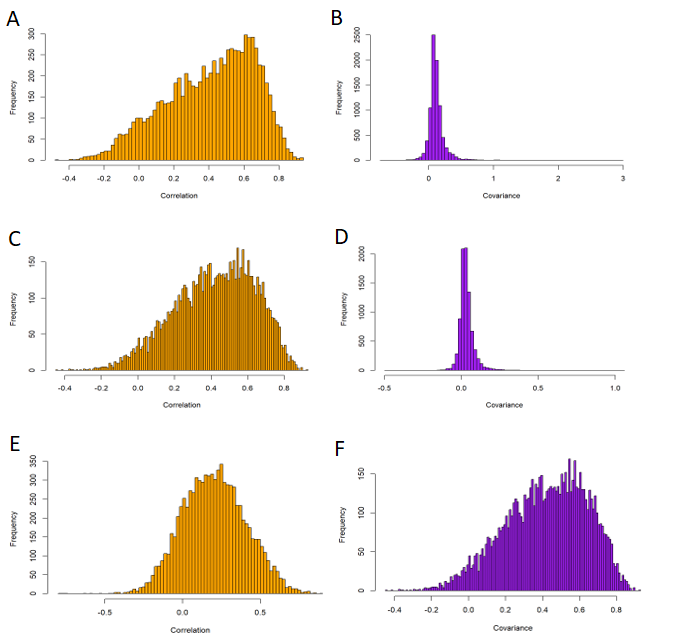

Supplement: Supplementary file 1 — Additional file 1. Supplementary Figures [file 12859_2019_3253_MOESM1_ESM.zip › figure1.png]

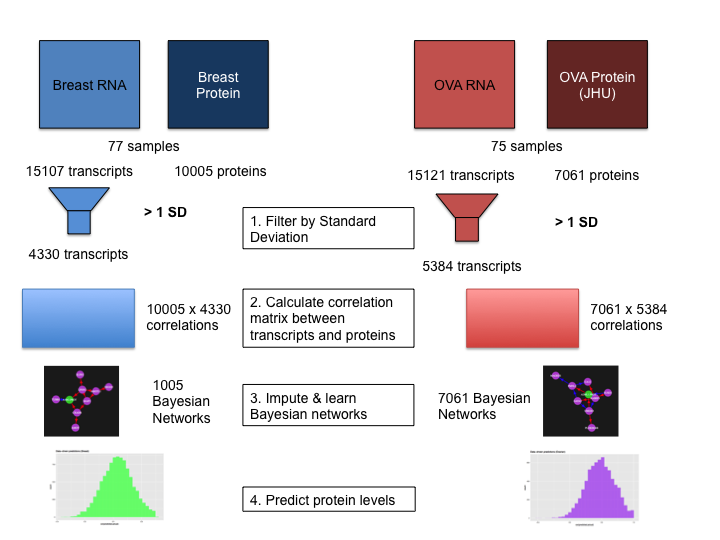

Supplement: Supplementary file 1 — Additional file 1. Supplementary Figures [file 12859_2019_3253_MOESM1_ESM.zip › figure2.png]

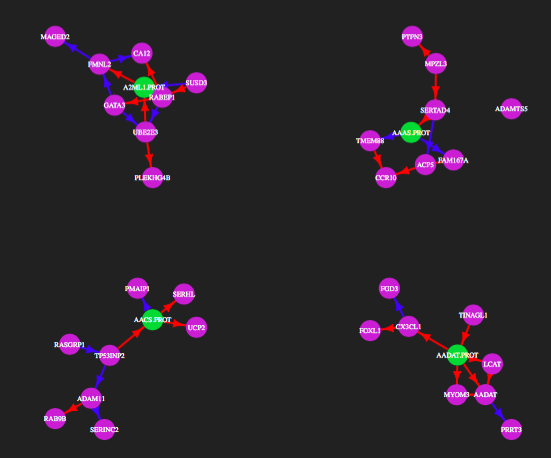

Supplement: Supplementary file 1 — Additional file 1. Supplementary Figures [file 12859_2019_3253_MOESM1_ESM.zip › figure3.png]

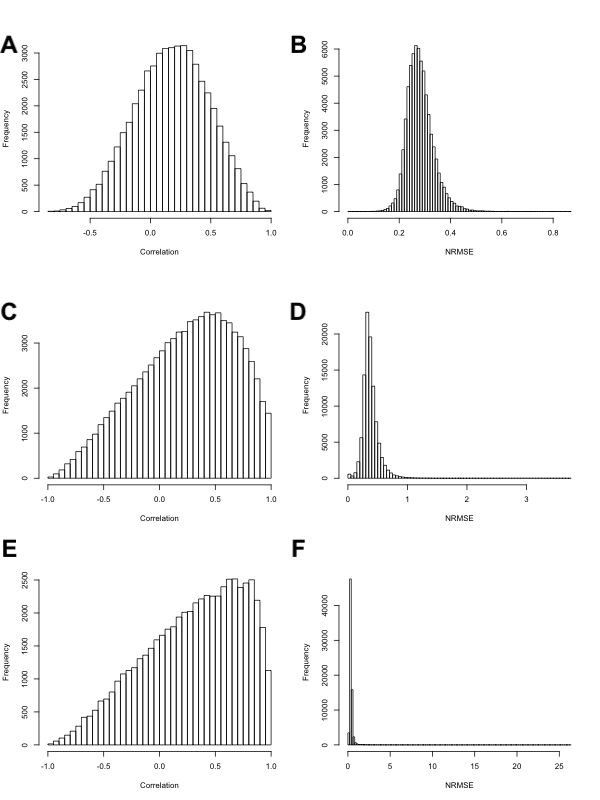

Supplement: Supplementary file 1 — Additional file 1. Supplementary Figures [file 12859_2019_3253_MOESM1_ESM.zip › figure4.png]

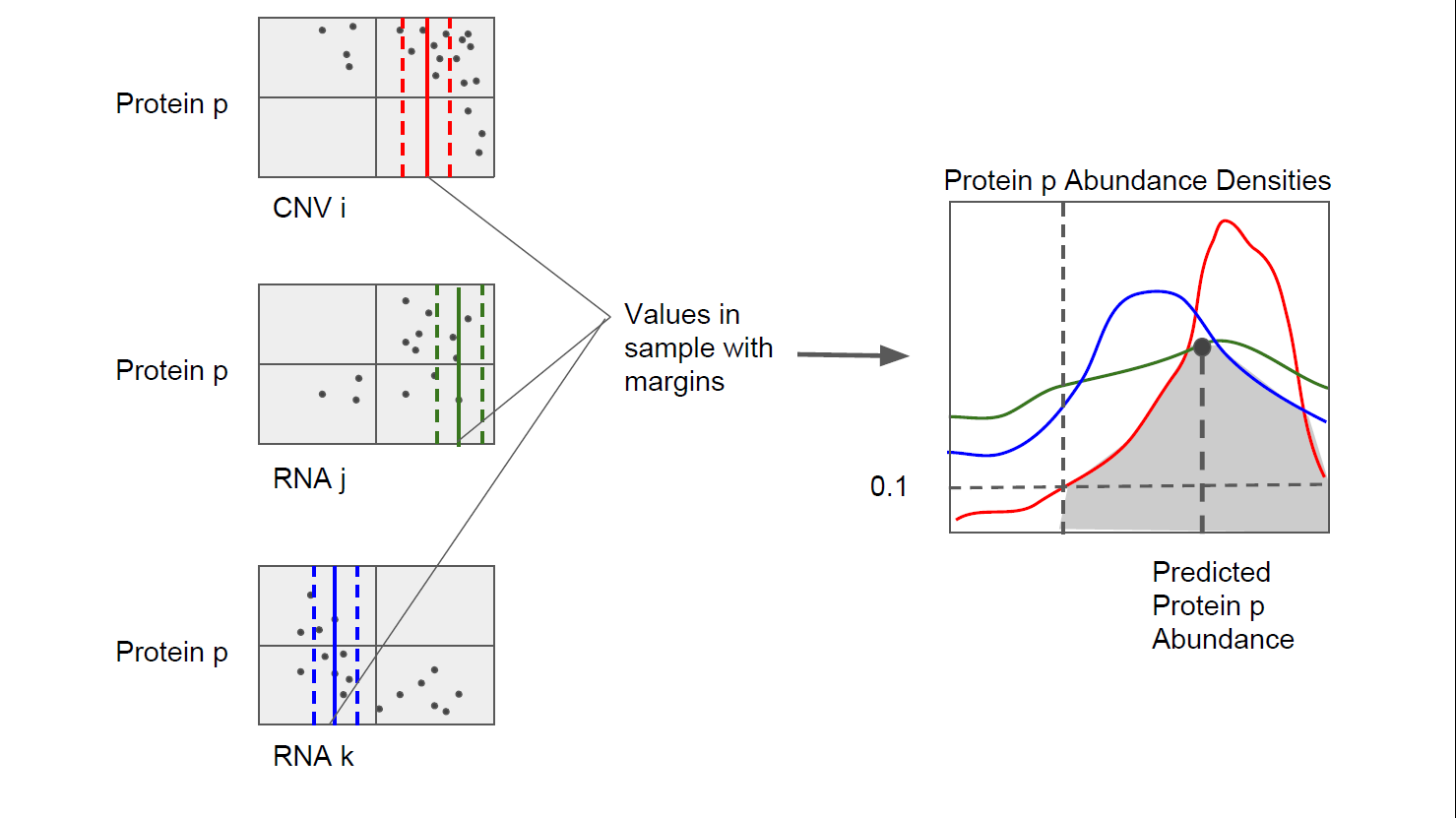

Supplement: Supplementary file 1 — Additional file 1. Supplementary Figures [file 12859_2019_3253_MOESM1_ESM.zip › figure5.png]

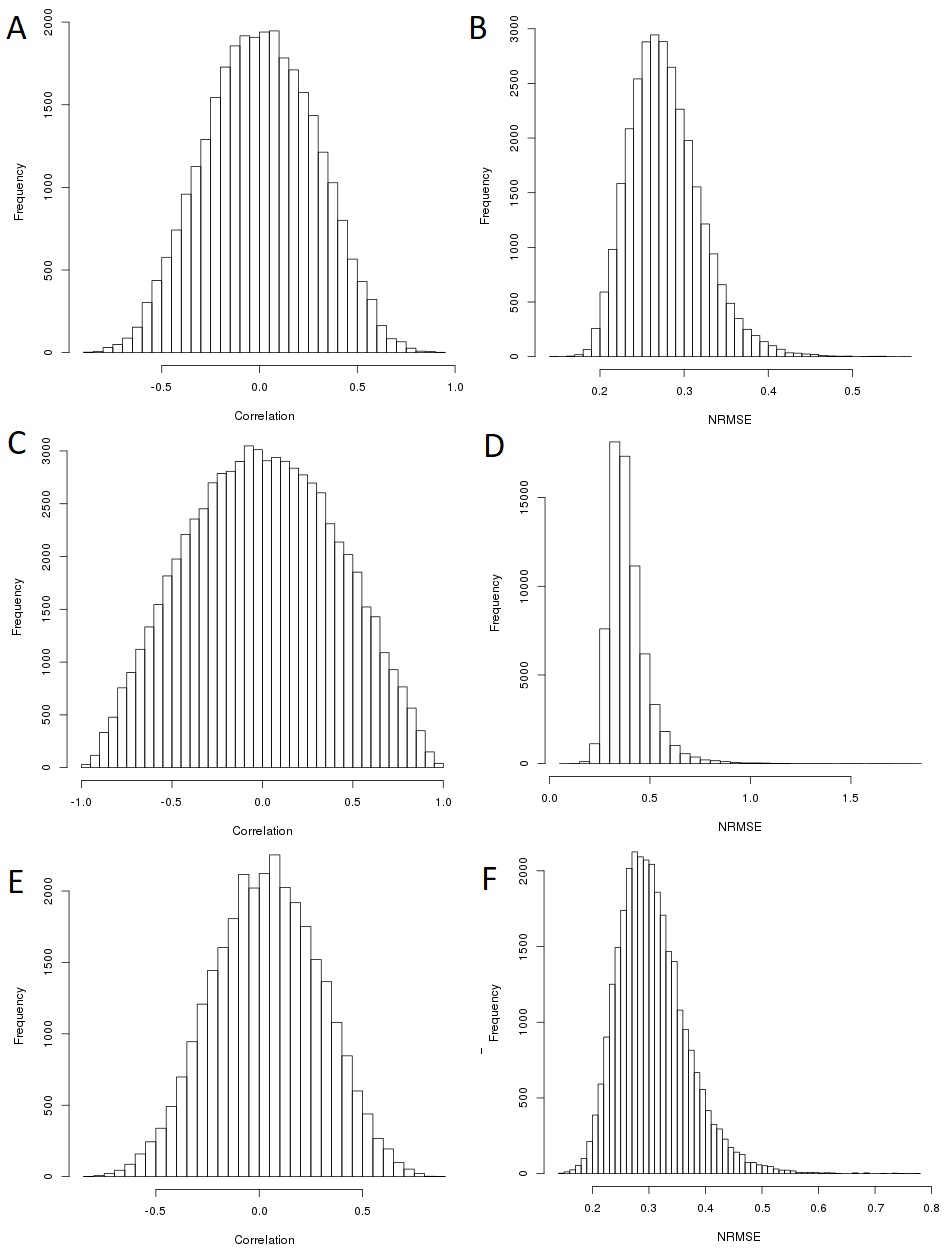

Supplement: Supplementary file 1 — Additional file 1. Supplementary Figures [file 12859_2019_3253_MOESM1_ESM.zip › figure6.png]

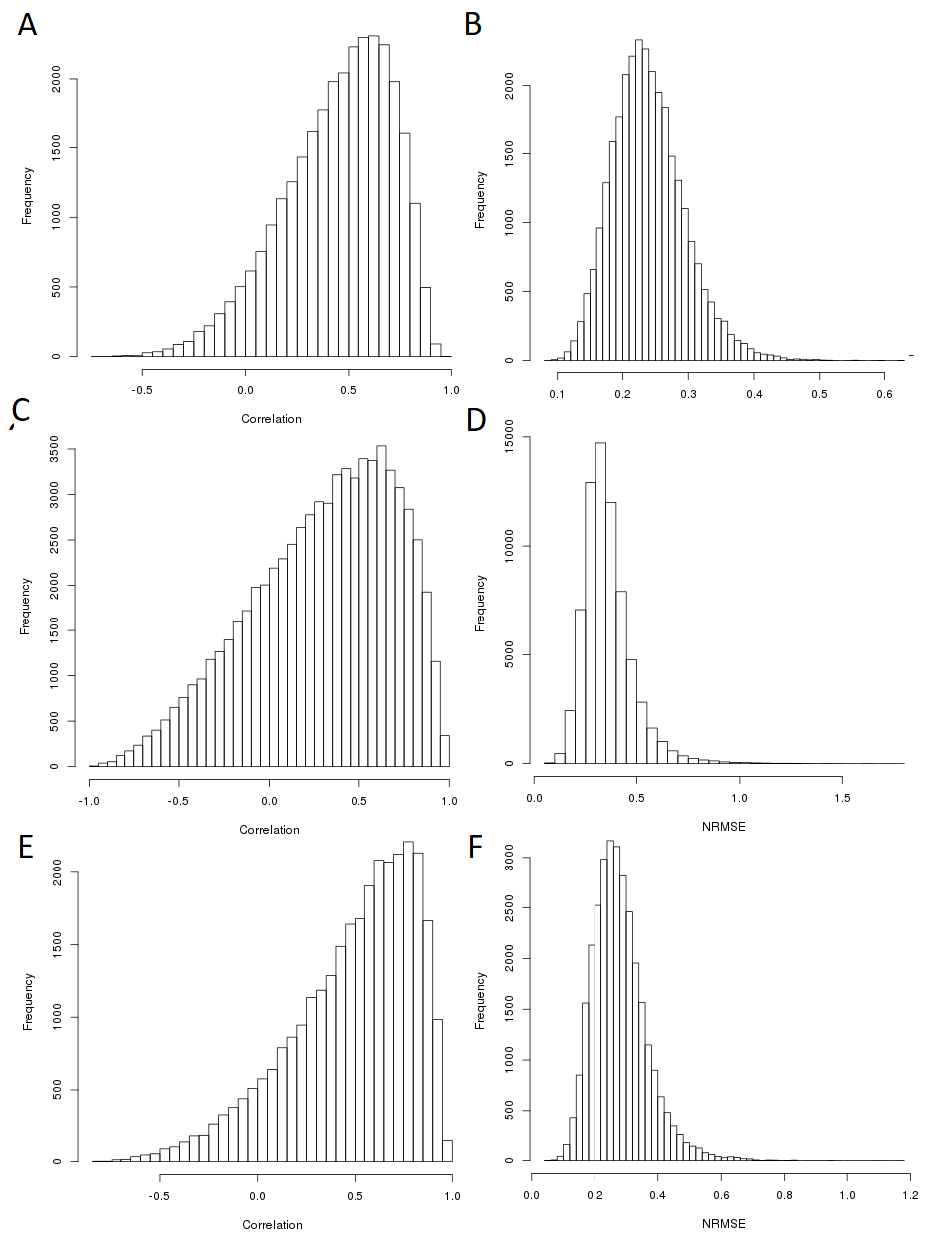

Supplement: Supplementary file 1 — Additional file 1. Supplementary Figures [file 12859_2019_3253_MOESM1_ESM.zip › figure7.png]
